# Molecular Aspects of Thyroid Calcification

**DOI:** 10.3390/ijms21207718

**Published:** 2020-10-19

**Authors:** Luciana Bueno Ferreira, Etel Gimba, João Vinagre, Manuel Sobrinho-Simões, Paula Soares

**Affiliations:** 1 Cellular and Molecular Oncobiology Program, Research Coordination, National Institute of Cancer, Rua André Cavalcante nº 37, Rio de Janeiro 20231-050, Brazil; etelgimba@id.uff.br; 2 Natural Science Department, Health and Humanities Institute, Fluminense Federal University, Rua Recife, Rio das Ostras 28895-532, Brazil; 3 Faculdade de Medicina da Universidade do Porto, Alameda Prof. Hernâni Monteiro, Porto 4200-319, Portugal; jvinagre@ipatimup.pt (J.V.); ssimoes@ipatimup.pt (M.S.-S.); psoares@ipatimup.pt (P.S.); 4 Cancer Signalling and Metabolism, Instituto de Investigação e Inovação em Saúde (i3S), Universidade do Porto, Rua Alfredo Allen 208, Porto 4200-135, Portugal; 5 Instituto de Patologia e Imunologia Molecular da Universidade do Porto (Ipatimup), Rua Júlio Amaral de Carvalho, Porto 4200-135, Portugal; 6 Departamento de Patologia, Centro Hospitalar Universitário de São João, Alameda Prof. Hernâni Monteiro, Porto 4200-319, Portugal

**Keywords:** calcifications, psammoma bodies, thyroid cancer, osteopontin

## Abstract

In thyroid cancer, calcification is mainly present in classical papillary thyroid carcinoma (PTC) and in medullary thyroid carcinoma (MTC), despite being described in benign lesions and in other subtypes of thyroid carcinomas. Thyroid calcifications are classified according to their diameter and location. At ultrasonography, microcalcifications appear as hyperechoic spots ≤ 1 mm in diameter and can be named as stromal calcification, bone formation, or psammoma bodies (PBs), whereas calcifications > 1 mm are macrocalcifications. The mechanism of their formation is still poorly understood. Microcalcifications are generally accepted as a reliable indicator of malignancy as they mostly represent PBs. In order to progress in terms of the understanding of the mechanisms behind calcification occurring in thyroid tumors in general, and in PTC in particular, we decided to use histopathology as the basis of the possible cellular and molecular mechanisms of calcification formation in thyroid cancer. We explored the involvement of molecules such as runt-related transcription factor-2 (Runx-2), osteonectin/secreted protein acidic and rich in cysteine (SPARC), alkaline phosphatase (ALP), bone sialoprotein (BSP), and osteopontin (OPN) in the formation of calcification. The present review offers a novel insight into the mechanisms underlying the development of calcification in thyroid cancer.

## 1. Introduction

Thyroid nodules (TNs) are defined by the American Thyroid Association (ATA) as “discrete lesions within the thyroid gland, radiologically distinct from surrounding thyroid parenchyma” [1]. They are extremely common and frequently identified in patients, with no symptoms, by self-examination or in undergoing evaluation for other medical conditions [2]. TNs may be discovered by palpation during a general physical examination or by radiographic exams, such as carotid duplex ultrasound (US), magnetic resonance imaging (MRI), computed tomography (CT) scans, or 18-fluorodeoxyglucose uptake on positron emission tomography scan (^18^FDG-PET) scanning. When detected in the latter exams, TNs do not correspond to palpable lesions and are therefore called as “thyroid incidentalomas” [3]. The method of identification determines the prevalence of TNs in the general population. When addressing palpation only, the prevalence ranges from 4 to 7% [4,5], whereas US detects nodules in 20–76% of the adult population [5,6], especially with the current use of high-resolution US techniques [2]. The incidence of malignancy detected in TNs is relatively low, ranging from 1.6 to 12% [7,8]. US is the primary tool for the diagnosis and the initial cancer risk stratification of TNs. Indeed, it guides decision making for fine-needle aspiration (FNA) biopsy, the subsequent clinical assessments at the time of long-term follow-up [9], and the eligibility for active surveillance of suspicious nodules [10]. US features evaluated in each nodule include echogenicity, composition (solid, cystic, mixed), margins, calcifications or other hyperechoic foci, shape, and relations with the thyroid capsule [11,12]. US patterns associated with malignancy comprehend hypoechogenicity; infiltrative, irregular, or lobulated margins; microcalcifications; taller-than-wide shape; and absence of a halo [13].

It was reported that 19.8–32.1% of TNs have some type of calcification [14,15] and that the prevalence of calcification in TNs is around 40% in malignant and 20% in benign nodules [16]. On the basis of Thyroid Image Reporting and Data System (TIRADS) scoring, microcalcifications are predictive of malignancy [16] and central macrocalcifications are usually predictive of benign pathology. Other diseases may be associated with calcifications, such as nodular goiter or Graves’ disease, and regardless of various studies on the topic, no clear association between calcifications and histopathologic classification has been demonstrated [17,18]. In contrast, microcalcifications in cervical lymph nodes are predictive of PTC metastasis [19].

The challenge to contribute to understanding the mechanisms involved in calcification in the field of thyroid pathology is to bridge the world of pre-operatory lesions with the world of real treatment. The occurrence of calcification in several types of thyroid lesions is easily identified and provides useful information regarding tentative diagnoses. After surgery, the precise description of morphological, immunohistochemical, and molecular features—which represent the so-called gold standard—allows for progress in the interpretation and reclassification whenever necessary. In other words, the pathologic meaning of the calcification in each thyroid lesion must be integrated in the specific context.

The objective of this review is to give a broad overview of the various facets within thyroid calcifications, encompassing details of molecular involvement. Our aim is to highlight the current understandings pertaining to the role of molecular components in modulating the calcification process.

## 2. Types of Calcification in Thyroid

Pathological calcifications can include dystrophic calcification, i.e., deposition of calcium at sites of cell injury and necrosis, and metastatic calcification, which refers to deposition of calcium in normal tissues caused by hypercalcemia (usually a consequence of parathyroid hormone excess); the latter will not be address in this review. The simplest way that thyroid nodular calcifications can be classified is according to their diameter and location. Under US, microcalcifications appear as hyperechoic (i.e., increased echogenicity relative to thyroid tissue) spots ≤ 1 mm in diameter with or without posterior acoustic shadows or as simple fine acoustic shadows [20]. They can be named as stromal calcification, bone formation, or psammoma bodies (PBs). Another type of small echogenic focus seen in the thyroid is inspissated colloid, which may cause a comet tail reverberation artifact in US [21,22].

Calcifications > 1 mm with posterior acoustic shadow are macrocalcifications, and although there are some different classifications for the types of macrocalcifications [23,24], the most commonly found terms are “egg-shell, annular or rim-like peripheral calcification” and “coarse dense calcifications” [16,25,26]. Regardless of size, all the aforementioned types of calcification represent forms of so-called dystrophic calcification (DC), since one is dealing with calcification occurring in degenerated or necrotic tissue.

**Figure 1:**
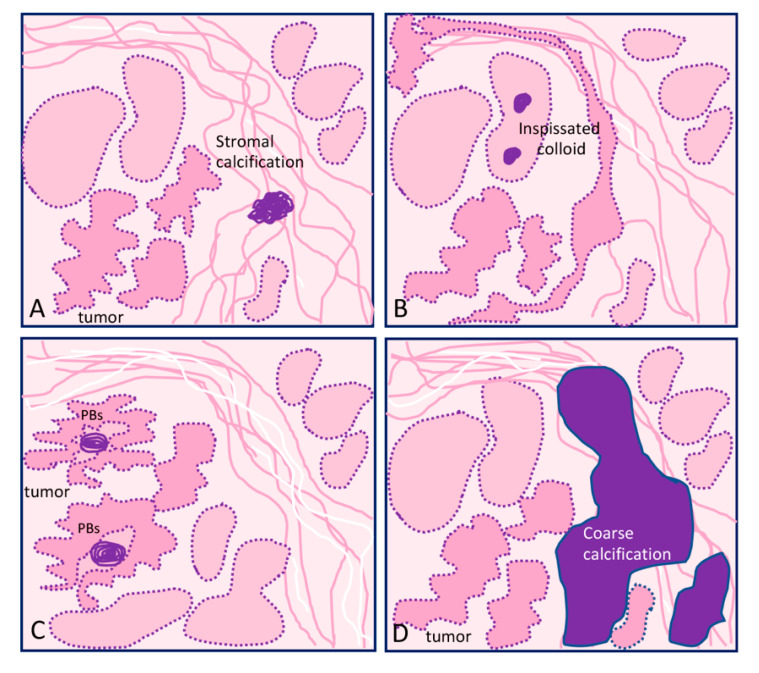
Graphical representation of the different types of calcification in thyroid tissue sections: (**A**) focus of stromal calcification (in purple color) in the tumor stroma, (**B**) inspissated colloid calcified, (**C**) psammoma bodies (PBs) (in purple color) located in the papillary thyroid carcinoma present inside lymphatic vessels or in the stalk of the papillae, and (**D**) coarse macrocalcification (in purple color). Shapes in pink correspond to non-tumor thyroid; shapes in deeper pink correspond to tumor thyroid.

The main types of calcifications are summarized in Table 1 and Figure 1. A more detailed description will be made for PBs, since they are more closely related with neoplastic transformation.

### 2.1. Microcalcifications: Psammoma Bodies (PBs)

**Figure 2:**
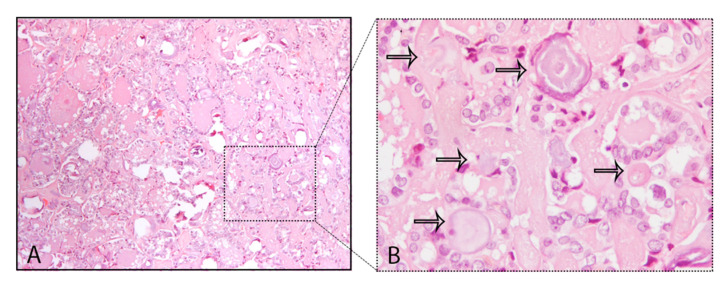
Psammoma bodies (PBs) in a papillary thyroid carcinoma: (**A**) visible PBs with purple color in hematoxylin and eosin (HE) staining, 10×; (**B**) magnified inset with PBs marked with the black arrows, 40×.

A common finding in thyroid are the calcifications known as PBs, sometimes designated as calcospherites. Most PBs are 50–70 μm round-shaped calcified concretions (Figure 1C). These structures present a glassy appearance, are concentrically laminated, and stain dark blue to black in Giemsa and purple in hematoxylin and eosin (HE) staining (Figure 2).

The main problem in histopathology, although not frequent, is to distinguish PBs from stromal calcification. PBs can usually be distinguished from granular calcium deposits associated with degeneration and from condensed colloid on the basis of typical concentric lamination, while the lack of birefringence distinguishes them from oxalate crystals often present in benign thyroid lesions [28]. The mechanism of PB formation in thyroid tumors remains controversial. Johannessen and Sobrinho-Simões [27] reported that they probably represent the end stage of two biologic events: (i) thickening of the basal lamina of the vascular stalk of the neoplastic papillae, followed by vascular thrombosis, calcification, and (possibly endothelial) cell necrosis, and (ii) intralymphatic necrosis and calcification of tumor thrombi in the thyroid adjacent to the main tumor or in the opposite thyroid lobe; these authors stressed that PB is an umbrella term covering several different entities that share light microscopic features that can be separated on electron microscopy. Johannessen and Sobrinho-Simões concluded that true ‘‘psammoma bodies’’ are formed by calcification of intravascular tumor thrombi or of infarcted tips of malignant papillae [27]. Cotran et al. [30] pointed that in the process of dystrophic calcification, single necrotic cells constitute seed crystals that become incrusted with the mineral deposits and the progressive acquisition of outer layers may create its lamellated configurations, giving rise to PBs. According to Majno et al. [31], dystrophic calcification has two major phases—initiation (nucleation) and propagation—which can occur intracellularly or extracellularly, whereas initiation of intracellular calcification occurs in the mitochondria of dead or dying cells. The initiators of extracellular calcification include phospholipids found in the membrane-bound vesicles, which are about 200 nm in diameter. Other authors [32] analyzed components of PBs in meningiomas and considered capillaries and degenerative cells as initiators of the formation of such calcareous bodies. Another possible mechanism refers to a humoral immune reaction—Tabuchi et al. [33] identified through immunohistochemistry the presence of immunoglobulin G (IgG)s in blood vessel whorls and in PBs in meningiomas, but there are no further studies corroborating these data. Several studies also reveal that the derivation and localization of PBs (within papillary cores, tumor stroma, or lymphatic vessels) is crucial in terms of context (site, morphology, and several important components for their definition) [27,34].

Besides thyroid, where PBs are found mainly in papillary thyroid carcinomas (PTCs) [24,35], meningiomas (45%) [36] and serous cystadenocarcinomas of ovary [37] also present PBs. PBs were reported rarely in other neoplasms, such as in insulinomas [38], lactotrope adenoma of the pituitary [39], serous papillary adenoma of borderline malignancy of the broad ligament, uterine serous carcinoma [40], endocervical adenocarcinoma [41], cholangiocellular carcinoma [42], chromophobe renal cell carcinoma [43], and in psammomacarcinoma of the peritoneum [44].

PBs are diagnostic only if clearly distinguished from coarse calcification and from inspissated colloid (Table 1) [45,46]. For that matter, coarse calcification is an irregularly shaped focus whereas inspissated colloid can present noncalcified colloid together with calcified bodies. Inspissated colloid can be found in a number of malignant or benign tumors, namely, Hürthle cell tumors, as well as non-neoplastic conditions including colloid goiter and Hashimoto’s thyroiditis [46,47,48,49]. Some tips can help in the distinction between PBs, namely, the location and context, the diagnosis of PTC, and the existence of inspissated colloid non-calcified together with inspissated colloid calcifications.

### 2.2. Macrocalcifications

Macrocalcifications may result from two pathologic processes. On one hand, degeneration of follicular cell lesions that leads to cystic formation due to infarction, hemorrhage, subsequent fibrosis, and occasionally calcification, with the latter being the final stage of scarring from a histopathological standpoint [50]. Macrocalcifications may be irregular in shape, and have been classified in different types according to the observations of the authors [16,26,51]. The two most commonly found classifications for macrocalcifications are described below and in Table 1.

#### 2.2.1. Eggshell, Annular, or Rim-Like Calcifications

This type of calcification in the TNs is commonly associated with benign nodules when it is complete. In contrast, uneven thickness or discontinuity of the calcification, it is suspicious for thyroid malignancy [52,53]. Focal interruption of an eggshell calcification can be explained by tumor infiltration through the broken calcification rim. Indeed, the presence of tissue outside the calcification should suggest malignancy and lead to a US-guided FNA [16].

#### 2.2.2. Coarse Macrocalcifications

Coarse macrocalcifications can also be referred to as dystrophic calcification and can be found in benign and malignant conditions of the thyroid including colloid goiters and anaplastic carcinomas (Figure 1D). On color or power Doppler US, a spoke-wheel vascular pattern centered on a coarse calcification is a strong argument in favor of benign follicular hyperplasia or thyroid adenoma [26]. Although a peripheral distribution can be seen in malignancy, the study results are conflicting and it is controversial if peripheral/rim coarse calcification has an increased malignancy risk [51].

The lack of a standard terminology and of a subclassification of the calcifications regarding morphologic features contributes to the absence of consensus for the importance of sonographically detectable calcifications. Several US categorization systems for echogenic foci in TN were developed, with some studies showing that echogenic foci previously termed as “microcalcifications” were present in benign nodules besides malignant TNs [21,22]. Tahvildari et al. [54] stressed that many authors referred to as “microcalcifications” that which do not exclusively represent PBs but rather other entities, including stromal calcifications and sticky or inspissated colloid. On the basis of the aforementioned data, the American College of Radiology Thyroid Imaging, Reporting and Data System (ACR TIRADS) proposed a terminology to describe echogenic foci in TN and has removed the word “microcalcification” from its lexicon and replaced it with more precise descriptors [17].

## 3. Calcification in Different Types of Thyroid Cancer

Among different types of thyroid carcinoma, calcification is mainly found in classic PTC and in MTC, although it has also been described in other thyroid carcinomas [54] (Table 2).

PBs within the thyroid gland are typically associated with classical PTCs, being found in up to 65% of cases [28,55,66,67]. In PTCs, microcalcifications representing true PBs are developed within the cores of papillae and/or in the tumor stroma and/or inside lymphatic vessels but often do not present calcification of neoplastic follicular colloid substance [68]. This explains why the follicular variant of PTC (FVPTC) has a very low frequency of microcalcifications (PBs) [69]. When FVPTC is infiltrative, the frequency of calcification can be higher but is rare in the encapsulated FVPTC [56,57]. Diffuse sclerosing variant of papillary thyroid carcinoma (DSVPTC) is an uncommon variant of PTC, characterized by diffuse involvement of one or both lobes of the thyroid gland [70]. This subtype also presents numerous PBs, so much that Koshikawa et al. [71] included “abundant psammoma bodies” in the list of cytological findings for DSVPTC. In MTC, calcification is a very common US finding and the reported incidences can vary from 20 to 54% [72,73].

## 4. Cellular and Molecular Mechanisms for Calcification in Thyroid Tissues

Mineralization is a biological process by which crystals of calcium phosphate (hydroxyapatite, HA) are deposited within the fibrous extracellular matrix (ECM) [74]. Physiological mineralization occurs in skeletal and dental tissues (bone, terminal hypertrophic cartilage, dentin, cementum, and enamel). This process can also occur ectopically (pathologic mineralization) in soft tissues, for instance in the blood vessels (arteriosclerosis calcification) or in joints during the late stages of osteoarthritis [75]. The progression and extent of both physiologic and pathologic mineralization are regulated locally and systemically. It also depends upon the availability of calcium and phosphate, concentration of mineralization inhibitors, and ECM composition [76].

Initiation of physiologic mineralization is facilitated by a specific population of extracellular vesicles (EV), named matrix vesicles (MVs), that were first discovered in chondrocytes and osteoblasts during bone tissue formation. They originate from the plasma membrane of mineral-forming cells (chondrocytes, osteoblasts, and odontoblasts), and their composition is different from that of the original cell plasma membrane, which may be related to MV mineralization [74]. These vesicles contain matrix-related enzymes, such as the metalloproteinases (MMPs) MMP-2, MMP-9, and MMP-13, which contribute to important roles in matrix remodeling, mainly through degradation of proteoglycans, allowing calcification [77,78]. MVs are composed of proteins and lipids that together support the accumulation of high concentrations of phosphate and calcium, as well as subsequent HA formation.

An important step in the mineralization process is the formation of the first crystal of HA, synthesized inside MVs by calcifying cells [79]. These MVs act as vehicles for the transfer of newly synthesized monocrystal from inside to the outside of the cell, forming a nucleation core of HA in the extracellular fluid [80]. The phenomenon of propagation of this monocrystal to appear as mature crystallized calcium remains poorly understood. However, it is thought that when HA crystals are exposed to the ECM, they serve as a template for the synthesis of the mature crystal [81]. 

Proteomic analyses of vesicles produced by chondrocytes, osteoblast cell lines, and bone marrow stromal cells undergoing osteogenic differentiation consistently detect numerous proteins that are involved in the mineralization process and matrix remodeling. This is in agreement with the notion that the biological function of MV is to support mineralization [82,83]. MVs are also enriched in Ca^2+^ transporters (annexins), which are commonly detected in many different types of EVs [84,85]. It has been discussed that although, much as with MVs, the calcifying EVs in the fibrillar collagen ECM of cardiovascular tissues serve as calcification foci, nevertheless, the formed mineral appears different between the tissues [86]. MVs are involved both in normal as well as in ectopic calcification. New et al. [87], reported that in atherosclerotic plaques, macrophages release MVs, which drive the formation of microcalcification. In thyroid, Tunio et al. [88] observed that cells present around PBs in PTC tissues were identified as CD68^+^ macrophages, suggesting that these macrophages may have contributed to the formation of PBs through release of MVs. Together, these findings indicate that MVs may play a pivotal role in pathophysiological mineralization of different organs/tissues [89] and that microcalcification in thyroid tumors may proceed through a similar process, i.e., mediated through MVs.

### 4.1. Runt-related Transcription Factor -2 (Runx-2)

Several lines of evidence demonstrated that the core binding factor-α1 (Cbfa 1)/runt-related transcription factor (Runx-2), a bone-specific transcription factor (TF), plays an important role in osteoblast differentiation and bone formation [90]. This TF stimulates the expression of osteoblast-specific genes, such as osteocalcin (OCN), type I collagen, and alkaline phosphatase (ALP), which induce osteoprogenitor cells to differentiate to osteoblasts [91]. Concerning non-osteoblastic cells, human smooth vascular cells undergo a spontaneous osteo/chondrocytic conversion and begin expressing Cbfa-1/Runx-2 in vitro [92], and it is hypothesized that progressive changes in the expression of genes encoding bone-associated proteins may be involved in the regulation of vascular mineralization.

The role of Runx2 in increasing the metastatic potential of tumor cells has been connected with its ability to regulate important genes related to tumor progression such as the vascular endothelial growth factor (VEGF) and osteopontin (OPN) [93,94]. It was shown that the regulation and transcriptional activity of Runx2 is linked to increased growth, invasion, and metastasis in breast, prostate, and colorectal cancers [95,96,97]. In thyroid carcinomas, Runx2 was upregulated in follicular cell-derived thyroid carcinomas and contributes to invasion and metastatic ability by regulating angiogenic/lymphangiogenic factors and epithelial mesenchymal transition (EMT)-related molecules. Runx2 increases the secretion of various MMPs to promote metastatic ability in thyroid cancer cells [98]. Carbonare et al. [99] demonstrated that Runx2 mRNA is overexpressed (7.81-fold expression) in pathological thyroid tissue in comparison with normal tissue. This study also showed that patients with microcalcifications expressed significantly higher levels of Runx2 mRNA in serum with respect to patients without microcalcifications. It was also reported by Jin et al. [100] that Runx-2 promotor activity was found to be enhanced by homeobox family A9 (HOXA9) that when overexpressed enhances ALP activity, calcification, and in vitro PTC cell line migration and invasion. Similarly, Endo et al. [101] found that overexpression of Runx-2 stimulated the expression of ALP, type I collagen, and OCN, as is the case in osteoblasts [91]. These results suggest a sequence of molecular events related to calcification, beginning with the overexpression of Runx-2, in PTC cells. Runx2 was also demonstrated to be involved in the regulation of EMT in thyroid carcinomas. Besides Runx-2 upregulation in PTC and in thyroid carcinoma cell lines, the authors reported its association with the mitogen-activated protein kinase kinase/extracellular signal-regulated kinase (MAPK/ERK) pathway. The silencing of Runx-2 down-regulates EMT-related molecules (snail family transcriptional repressor (SNAI)2, SNAI3, and twist-related protein 1 (TWIST1)), MMP2, and vasculogenic factors (VEGFA and VEGFC) in thyroid carcinoma cells, and suppresses thyroid carcinoma cell invasion in transwell assays [98]. Another gene associated with Runx-2 and calcification is Galectin-3 (Gal-3). Gal-3 is a member of the lectin family and plays an important role in cell–cell adhesion and cell–matrix interactions. Kaptan et al. [102] revealed that regulation of Gal-3 expression was strongly correlated with Runx2 TF in human thyroid carcinoma; increase in Gal-3 gene expression was detected in patients with calcification [103].

Komori et al. [104] reported that disruption of Runx-2 results in a complete lack of bone formation. These data clearly demonstrate that Runx-2 plays a key role in osteogenesis. OCN, another bone-specific gene, was also expressed in these cells and malignant tissues. The promotor region of OCN is known to be a target for Runx-2. A non-spliced form of OCN transcript has been reported in several non-osseous tissues [105] such as the prostate, skeletal muscle, ovary, and thyroid. In contrast, the major OCN transcripts in papillary thyroid cell line (BHP18–21) had no intronic sequences, indicating that they were the mature bone type [101]. A study by Gadeau et al. [106] suggested that OCN is not involved in the initiation steps of the calcification processes. In a rabbit model of injured aorta, they showed that this protein is not detected in early calcifications. In contrast, it is present in late calcium deposits, suggesting that OCN might be involved in the control of calcification rather than in its genesis. In a previous work, we demonstrated that the levels of OCN increase over the weeks (1 and 2 weeks) of culturing papillary thyroid carcinoma cell line (TPC1) while mineralizing the extracellular matrix [107].

### 4.2. Secreted Protein Acidic and Rich in Cysteine (SPARC)

Osteonectin, also called SPARC (secreted protein acidic and rich in cysteine) or basement-membrane protein 40 (BM-40), is a matricellular protein, an important mediator of tumor cell progression, and has been implicated in a variety of diverse biological processes, including cell adhesion, proliferation, angiogenesis, tumor cell migration, and invasion [108]. It is an important protein expressed by the juxtatumoral stromal cells in infiltrating breast carcinoma [109]. In thyroid cancer, this protein was found to be overexpressed in malignant tumor subtypes when compared to benign subtypes [110]. Takano et al. [111] showed that increased expression of SPARC was observed in all histological types of thyroid tumors analyzed, especially in anaplastic carcinomas. However, the expression levels of osteonectin in cell lines derived from anaplastic carcinomas were almost the same as those in differentiated tumors. The authors speculated that the increased expression of this protein in anaplastic carcinomas may not be due to its overexpression on tumor cells but by the stromal fibroblasts or vascular endothelial cells, as observed in colon and hepatocellular carcinoma.

SPARC was also related to the phenotype of macrophages. Toba et al. [112] demonstrated that SPARC treatment in mice increased expression of proinflammatory macrophage M1 markers. Remarkably, it was demonstrated that polarization of macrophage influences the process of calcification in human aortic valve. The shift toward M1 phenotype might promote valve interstitial cell calcification [113].

### 4.3. Alkaline Phosphatase (ALP)

Alkaline phosphatase (ALP) enzymes are encoded by distinct genes as many tissue-specific isozymes. This is one of the first functioning genes in the calcification mechanism. ALP is produced early in growth and is easily found on the surface of the cell and in MVs of all tissues, including bones and calcifying cartilages [114], whereas when some genes are upregulated, such as OCN, the ALP expression diminishes. Opposite to OCN, ALP must function in the initial phases of the mineralization process. The mechanisms through which ALP expression is regulated is being investigated [114]. Two steps are accompanied by mineralization. In a first step, it starts with hydroxyapatite (HA) crystal formation in the MVs and then it follows with the distribution of HA to the ECM via the membrane. Annexins create a calcium channel in the MVs and introduce calcium into the vesicle layer. The aggregation of calcium in MVs is facilitated by calcium-binding phosphatidylserine, calcium-binding proteins such as calbindin D9k, and bone sialoprotein (BSP) [115,116]. A high expression of ALP in breast cancer has been reported. Mineralizing cell lines (MDA-MB-231 and SKBR3) displayed higher levels of ALP activity that was further increased by the addition of mineralization-promoting media [117]. Additionally, elevated levels of ALP in the serum of breast cancer patients were detected when compared to controls [118], in patients with bone metastases in comparison to patients without bone metastases [119,120], and also in advanced stages of breast cancer as compared to early stages and/or healthy controls [121,122]. These data suggest that in certain circumstances, a subpopulation of epithelial breast cancer cells may switch to osteoblast-like cells.

### 4.4. Bone Sialoprotein (BSP)

BSP is a member of the SIBLING (small integrin-binding ligand N-linked glycoprotein) family. BSP is overexpressed in many malignant tissues, including breast, prostate, and thyroid carcinomas [123]. Its expression has been associated with clinical severity and poorer survival among patients with breast cancer [124]. Evaluated by immunohistochemistry, this bone matrix protein was detectable in 72% of thyroid carcinomas studied by Bellahcene et al., including PTC, MTC, follicular thyroid carcinoma (FTCs), poorly differentiated thyroid carcinoma (PDTCs), and anaplastic thyroid carcinoma (ATC) subtypes. In this study, FTCs expressed lower levels of BSP when compared to PTC, and microcalcifications were usually observed in PTCs associated with tumor areas with high levels of BSP. In bone, several studies provide evidence that BSP is implicated in the mineralization process [125] and this glycoprotein was shown to act as a nucleator of HA in vitro [126]. The authors hypothesized that the high amounts of BSP expressed by thyroid carcinoma cells could be responsible for the ectopic formation of calcified deposits in these tumors [127]. Wu et al. [128] evaluated protein levels of BSP in PTC samples by immunohistochemical analyzes and correlated it with the levels of another bone matrix protein, OPN. The authors found a significant difference in the immunohistochemical score for BSP and OPN protein staining between PTC specimens with and without calcification, and also the level of BSP protein staining was found to be significantly correlated with the level of OPN protein staining in PTC specimens. They conclude that the strong correlation between BSP and OPN in PTC suggests a role for BSP and OPN in calcification and tumor progression of PTC.

### 4.5. Roles of OPN in Mineralization and Related-pathological Conditions

Another important modulator of thyroid mineralization and calcification is OPN, a non-collagenous calcium-binding glycophosphotein, rich in sialic acid, with structural and functional characteristics of a matricellular protein. OPN binds tightly to hydroxyapatite and seems to form an integral part of the mineralized matrix [129]. OPN was the first ECM protein identified in the bone tissue [130] and a member of a family of phosphorylated sialoproteins of mineralized connective tissues [131]. Some structural and biochemical features endow OPN properties to efficiently bind to calcium and to interact with high affinity to hydroxyapatite crystals. Phosphoproteins, such as OPN, have been widely linked to the mineralization process on the basis of their accumulation at the mineralization sites. In addition, the inefficacy of dephosphorylated bone matrices to support mineralization has been shown [131,132]. OPN contains several conserved structural elements, including heparin- and calcium-binding domains, a thrombin-cleavage site, a cluster of differentiation 44 (CD44)-binding site, and two integrin-binding sites [133], some of them importantly related to mineralization properties. The arginine/glycine/aspartate (RGD) adhesive domain is recognized by several integrins, and was shown in vitro to serve as an attachment substrate to several cell types, such as osteoclasts, primarily via the αVβ3 integrin [134]. OPN is a ligand for the cell membrane receptor CD44v6, required for efficient OPN binding. Other two conserved N-terminal domains with heparin binding homology possibly regulate OPN binding to other ECM macromolecules related to tissue mineralization to form supramolecular structures. OPN has been demonstrated to bind directly to fibronectin, collagen, and osteocalcin [135,136].

Due to these diverse features, including OPN properties as a cell adhesive and signaling molecule for various tissue microenvironment cells, in addition to being a robust regulator of osseous and ectopic calcification, this protein is a key modulator of mineralization in several tissues, including the thyroid [137]. OPN is released in mineralized tissues, including bones and teeth, and produced by osteoclastic and osteoblastic cells at high levels acting in bone structuring. OPN expressed from osteoclasts is known to inhibit the hydroxyapatite, indicating that OPN is associated with bone destruction [138]. In the kidney, OPN inhibits calcium oxalate crystal formation and retention [139]. In line with such data, OPN has a significant importance in the suppression of ectopic calcification and over-mineralization [130] and takes an active role in wound healing and preventing renal stone formation [131]. In the kidney, OPN regulates renal nitric oxid synthase (iNOS) and urinary calcium oxalate deposition [140]. OPN is correlated with calcium deposition and mineralization on several pathological conditions, including cancer [141]. OPN and BSP are bone matrix proteins that have been implicated in the selective affinity of cancer cells for bone [142], with this potentially depending on the direct involvement of bone matrix.

Ectopic OPN expression may define a critical and initial event in the calciphylaxis pathogenesis, which represents a unique calcific thrombogenic vasculopathy [142]. OPN may also play an important role in stone formation within the pancreatic duct system in chronic pancreatitis [143]. It has been reported that there is a close relationship between hydroxyapatite crystals and OPN-producing histiocytes in breast cancer tissues, suggesting that OPN plays a role in the biomineralization that occurs in certain noninvasive breast cancers and atypical cystic lobules [144]. The expression of OPN is related to calcification and to the development of breast cancer tissues [145]. OPN expression level is also significantly correlated with the degree of calcification, besides being involved as a core protein in the formation of craniopharyngioma calcification [129], and is associated with the stromal calcification in lung neuroendocrine carcinoma (LCNEC) tumors [146]. Other authors have described the association between OPN and cell matrix calcification in meningioma tissues, in which OPN protein is co-localized with calcium phosphate deposits, further evidencing a role for OPN on the PB formation in this context [147].

Specifically, in thyroid carcinomas, OPN mRNA-expressing cells have been found around the PBs, and the localization of OPN protein was found to be consistent with that of these structures. The OPN mRNA-expressing cells were identified as CD68^+^ macrophages and OPN secreted by these cells may play a significant role in the development of PBs in PTCs [88]. OPN expression is significantly correlated with microcalcification and lymph node metastasis in PTC tissue samples, further evidencing a possible role for OPN in the formation of microcalcification in this tumor type [148]. Other authors proposed that OPN plays an important role in the molecular mechanisms related to calcification in PTC [149] and that there was also a significant correlation of OPN protein levels and calcification in these tumor types [128]. More recently, our group revealed that OPN expression was associated with the presence of PBs in the classical variant of PTC (cPTC). We found that cPTC samples presenting PBs showed higher OPN expression levels. In TC cell lines, OPNa splice variant overexpression promoted higher matrix calcification and collagen synthesis when compared to the patterns observed for two other OPN splice variants (OPN-SV), named OPNb and OPNc. In response to OPN knockdown, calcification was inhibited, paralleled with the downregulation of calcification markers such as collagen and osteocalcin [107]. Hence, our data indicated that OPN expression is associated with the presence of PBs in PTC samples and demonstrated that among the OPN-SV, OPNa, besides activating cell migration and invasion in TC cell lines [150], is the main contributor to calcification in tested TC cell lines, providing further indications to a better understanding of the biology and the etiopathogenesis of the calcification process in TC cells [107]. However, our data contrasts with the previously described roles of OPN as an inhibitor of calcification, namely, in bones [138] and in vascular calcification [130]. OPN typically inhibits growth of calcium-containing crystals in models of bone mineralization and nephrolithiasis [151]. Similar effects were demonstrated in a gel-based model of hydroxyapatite crystal formation [132]. Otherwise, fewer studies support a stimulatory role for OPN in mineralization [152]. For instance, calcium pyrophosphate dihydrate (CPPD) crystals are commonly found in osteoarthritic joint tissues and it was reported that OPN stimulates ATP-induced CPPD crystal formation by chondrocytes in vitro. Thus, OPN may play an important role in facilitating CPPD crystal formation in articular cartilage [153]. OPN was reported as an activator of calcification in dental pulp [154] and craniopharyngioma calcification [129], as well as in the calcification process of some tumor types, such as in breast cancer and LCNEC tumors. On the basis of these data, it is speculated that in the thyroid tumor cell context, OPN may behave as a positive modulator of matrix calcification, as proposed by others, in which OPN may be involved in the control of calcification rather than its genesis [155]. A common explanation for the variable OPN effects over mineralization and calcification is related to the OPN’s vast post-translational modifications (PTM). Osteopontin contains sites for several PTM, such as phosphorylation, glycosylation, transglutamination, and thrombin cleavage, which can favor OPN binding to other proteins related to mineralization process as well as to the matrix constituents [156]. The OPN phosphorylation patterns and its solubility state in relation to matrix components may be key factors that can modulate OPN effects in mineralization models [157].

## 5. Conclusion/Future Prospects

**Figure 3:**
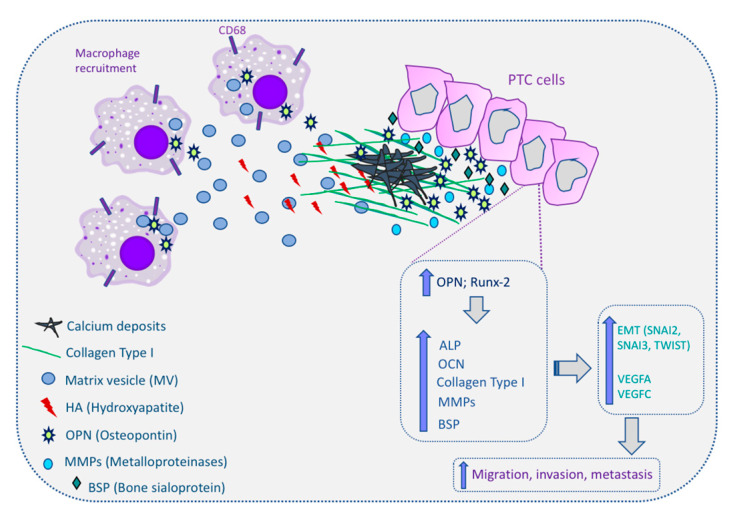
Molecular mechanism of calcification in papillary thyroid cancer (PTC) cells. Macrophages can be recruited to the PTC microenvironment and release matrix vesicles (MVs) to the extracellular matrix (ECM). MVs contain hydroxyapatite (HA), which initiates the calcification process. Osteopontin (OPN) and runt-related transcription factor-2 (Runx-2) are overexpressed in PTC cells and this increases the expression of alkaline phosphatase (ALP), osteocalcin (OCN), collagen type I, metalloproteinases (MMPs), and bone sialoprotein (BSP). All these molecules are involved in the induction of calcium deposits in the ECM, culminating in the calcification process in thyroid tissues, and also induce the expression of epithelial–mesenchymal transition genes (snail family transcriptional repressor (SNAI)2, SNAI3, and twist-related protein 1 (TWIST1)) and angiogenic factors (vascular endothelial growth factor (VEGF)A and VEGFC). Up arrows mean increase; The cells expressing CD68 correspond to macrophages.

Thyroid cancer, in particular PTC, presents distinct types of calcification processes, notably the psammoma bodies. The role and significance of these calcifications for thyroid cancer diagnosis and prognosis has been explored in several studies but without conclusive indications. Accepting the adjuvant role of micro-calcifications in the imagological and cytological diagnosis of PTC, its significance in tumor aggressiveness, including metastatic behavior of PTC, remains controversial. Overall, the process by which calcification occurs in thyroid cancer remains poorly understood. However, all aforementioned data relating several dysregulated molecules with the calcification process in thyroid provides some clues and improves the understanding of the molecular mechanisms of microcalcification in thyroid cancer as well as in the tumorigenesis. The proposed molecular mechanism for calcification in papillary thyroid cancer are summarized in Figure 3.

## Figures and Tables

**Table 1:** Main types of calcification found in thyroid lesions.

	Localization	Type of Lesion	Description
**Microcalcification ≤1 mm**			
Psammoma bodies (PBs)	Inside lymph vessels or in the papillae axis	True PBs Classical PTC	50–70 μm round-shaped, concentrically laminated, calcified concretions with a glassy appearance (Figure 1C).
Inspissated colloid calcified	Inside follicles	False PBs Benign nodules	Thick colloid (colloid crystals) can present microcalcifications over inspissated colloid and lead to focal hyperechogenic foci; can potentially be confused with PBs (Figure 1B).
Stromal microcalcification	Around follicles	False PBs	Spherical crystalline bodies with a diameter of 0.1–2.5 μm. Usually too small to be detected by light microscopy and apparently arise within basal laminae as a result of concentric deposition of calcium salts or calcifications of membrane-bound vesicles. Calcified collagen fibrils can rarely be observed [27,28] (Figure 1A).
Bone calcification	Connective tissue	False PBs	Bone formation is considered when there is both bone matrix and osteocytes in the connective tissue of a thyroid nodule, regardless of being neoplastic or not [29,30]
**Macrocalcification >1 mm**			
Eggshell, annular, or rim-like calcifications		Benign and malignant lesions	Annular or rim-like peripheral calcification, defined in US as curvilinear hyperechoic structures parallel to the margin of the nodule.
Coarse calcifications	Stroma	Benign and malignant lesions	An irregularly shaped focus of calcification (can comprise micro- and macrocalcifications) (Figure 1D).

**Table 2:** Main types of calcification present in thyroid lesions.

Calcification (micro/macro)	Tumor Subtype
Micro- and macrocalcifications [28,55]	classical/conventional PTC
Microcalcifications [56,57]	infiltrative follicular variant of PTC
Microcalcifications [58] Scattered microcalcifications [59,60]	diffuse sclerosing variant of PTC
Lymph node involvement with nodal microcalcifications [59,60]	macrofollicular variant of PTC
Micro- and macrocalcifications [61]	Hürthle cell carcinoma
Microcalcifications [59]	hobnail variant of PTC
Microcalcifications [62] Macrocalcifications [63]	hobnail variant of PTC
Macrocalcification [64,65]	Hürthle cell tumors
Micro and macrocalcifications [23]	MTC

PTC: papillary thyroid carcinoma; MTC: medullary thyroid carcinoma.

## Abbreviations

ATA	American Thyroid Association
ACR	American College of Radiology
ACCP	Amorphous carbonated calcium phosphate
ALP	Alkaline phosphatase
BSP	Bone sialoprotein
CA	Carbonated calcium apatite
Cbfa 1	Core binding factor-1
CCL	Chemokine ligand
CD44	Cluster of differentiation 44
CPPD	Calcium pyrophosphate dihydrate
CT	Computed tomography
DC	Dystrophic calcification
DSVPTC	Diffuse sclerosing variant of papillary thyroid carcinoma
ECM	Extracellular matrix
EMT	Epithelial–mesenchymal transition
EV	Extracellular vesicles
FNA	Fine needle aspiration
FTC	Follicular thyroid cancer
FVPTC	Follicular variant of papillary thyroid carcinoma
Gal-3	Galectin-3
HA	Hydroxyapatite
HOXA9	Homeobox family A9
IgG	Immunoglobulin G
iNOS	Nitric oxid synthase
LCNEC	Large-cell neuroendocrine carcinoma
LPV	Leucine/proline/valine
M1	Macrophage type 1
MAPK/ERK	Mitogen-activated protein kinase kinase/ extracellular-signal-regulated kinase
M-CSF	Monocyte colony stimulating factor
MMP	Metalloproteinase
MTC	Medullary thyroid carcinoma
MV	Matrix vesicles
MVFPTC	Macrofollicular variant of papillary thyroid carcinoma
MRI	Magnetic resonance imaging
mRNA	Messenger ribonucleic acid
OCN	Osteocalcin
OPN	Osteopontin
OPN-SV	Osteopontin splice variants
PB	Psammoma bodies
PTC	Papillary thyroid carcinoma
PTM	Post-translational modification
RGD	Arginine/glycine/aspartate
Runx-2	Runt-related transcription factor-2
SIBLING	Small integrin-binding ligand N-linked glycoprotein
SNAI	Snail family transcriptional repressor
SPP1	Secreted phosphoprotein 1
SVVYGLR	Serine/valine/valine/tyrosine/glutamic acid/leucine/arginine
SPARC	Secreted protein acidic and rich in cysteine
TAM	Tumor-associated macrophage
TF	Transcription factor
TN	Thyroid nodule
TNF	Tumor necrosis factor
TIRADS	Thyroid Image Reporting And Data System
TWIST	Twist-related protein
US	Ultrasound
VEGF	Vascular endothelial growth factor
